# Early Blockade of TLRs MyD88-Dependent Pathway May Reduce Secondary Spinal Cord Injury in the Rats

**DOI:** 10.1155/2012/591298

**Published:** 2012-05-22

**Authors:** An-hui Yao, Li-yun Jia, Yu-kai Zhang, Quan-rui Ma, Peng Cheng, Ling Liu, Gong Ju, Fang Kuang

**Affiliations:** ^1^Institute of Neurosciences, The Fourth Military Medical University, 17 West Changle Road, Xi'an 710032, China; ^2^Department of Human Anatomy, Ningxia Medical University, 1160 Victory Street, Yinchuan 750001, China

## Abstract

To determine the role of toll-like receptors (TLRs) myeloid differentiation factor 88 (MyD88) dependent pathway in the spinal cord secondary injury, compression injury was made at T8 segment of the spinal cord in adult male Sprague-Dawley rats. Shown by RT-PCR, TLR4 mRNA in the spinal cord was quickly elevated after compression injury. Intramedullary injection of MyD88 inhibitory peptide (MIP) resulted in significant improvement in locomotor function recovery at various time points after surgery. Meanwhile, injury area, p38 phosphorylation, and proinflammation cytokines in the injured spinal cord were significantly reduced in MIP-treated animals, compared with control peptide (CP) group. These data suggest that TLRs MyD88-dependent pathway may play an important role in the development of secondary spinal cord injury, and inhibition of this pathway at early time after primary injury could effectively protect cells from inflammation and apoptosis and therefore improve the functional recovery.

## 1. Introduction

Acute traumatic spinal cord injury (SCI) is an unexpected, catastrophic event which causes various lifelong disabilities of the patients. The consequences also result in grave influences in a row to family members and society. However, there are no fully restorative therapies for SCI up to now [1–3]. The pathological sequelae following acute SCI are divided into two phases: primary mechanical injury and secondary injury [4–6]. Primary SCI is caused by direct mechanical trauma, and it instigates a progressive wave of secondary injury via activation of a series of pathophysiological mechanisms including alterations in microvascular perfusion, inflammation, lipid peroxidation, free radical generation, apoptotic/necrotic cell death, and dysregulation of ionic homeostasis [7–11]. These mechanisms caused the destruction of axonal tracts that were left intact after the initial trauma, which is the major impediment to functional recovery after SCI. It has been proved that fibers spared as few as 5–10% in the spinal cord are sufficient to facilitate basic locomotion recovery following SCI in rats [12]. Therefore, how to protect the spared fibers and reduce the secondary injury has become the top priority for scientists working in the research of SCI. 

Inflammatory responses are very important, even central, in the pathological process of the acute and chronic phases of secondary injury. During the secondary injury phase, the central nervous system (CNS) evokes both innate and adaptive immunities [11, 13]. After primary SCI, resident microglia, invaded macrophages and dendritic cells could work as antigen presenting cells through toll-like receptors (TLRs) signaling [14] at and around the injury site. At least 12 TLRs have been identified in the mammals, and they are involved in recognition of pathogen-associated molecular patterns (PAMPs) and activation/regulation of both innate and adaptive immunities [15, 16]. Moreover, it was recently shown that some TLRs (e.g., TLR2, TLR4) can be stimulated by endogenous molecules named danger-associated molecular patterns (DAMPs) [1]. DAMPs could be released from injury sites of the tissue [17, 18]. In SCI, many DAMPs including heat-shock proteins (HSPs), necrotic cells, fibronectin and hyaluronic acid, heparan sulphate, lung surfactant protein A, high-mobility group box 1 and mRNA would be increased at sites of spinal injury, as accumulated studies reported [19–26]. Meanwhile, research with genetic deficient mice has indicated that TLR2 or TLR4 deficiency impaired function recovery in SCI [27], while research with TLR4 loss-of-function mutation mice showed that TLR4 deficiency protected against focal cerebral ischemia and axotomy-induced neruodegeneration [28]. 

The signaling pathways activated by TLRs are broadly classified into MyD88-dependent and -independent pathways because MyD88 is the universal adapter protein recruited by all TLRs except TLR3 [29, 30]. TLRs MyD88-dependent pathway activates nuclear-factor-*κ*B (NF-*κ*B) [31] and subsequently results in the production of inflammatory cytokines (e.g., TNF-*α*, IL-1*β*) [32–35], while MyD88-independent pathway is related to transcriptional activation of type I interferons [36–38] and also activates late phase NF-*κ*B via TAK which is shared by MyD88-dependent pathway. However, the exact roles of these pathways in the pathophysiology of SCI remain unclear. 

NF-*κ*B is essential for neurons survival against oxidative stress and ischemic degeneration [39], but it also contributes to inflammation and apoptosis after CNS injury [40, 41]. The different phases of NF-*κ*B may serve distinct role in this balance. Therefore, we hypothesized that TLRs MyD88-dependent pathway correlated with the development of spinal cord secondary injury via inflammatory reaction. In this study, at first we detected TLR4 mRNA expression by RT-PCR in the spinal cord at several time points after compression SCI, then used inhibitory peptide to block MyD88-dependent signaling at early time after SCI, evaluated motor functional recovery by Basso-Beattie-Bresnahan (BBB) score, beam-walking test, and foot print analysis, and investigated histological damage, cell apoptosis, and inflammatory reactions with immunohistochemistry (IHC) and Western blotting assay. We found that inhibition of MyD88-dependent pathway at early time could reduce the inflammatory response, preserve more neurons, reduce the lesion size in the spinal cord, and therefore promote motor functional recovery from spinal cord compression injury.

## 2. Materials and Methods

### 2.1. Animals and Surgical Procedure

Male Sprague-Dawley rats (200–220 g) were purchased from Experimental Animal Center of the Fourth Military Medical University. Rats were housed in a 12/12 h light/dark cycle room with standard rodent food and water available *ad libitum*. Efforts were made to minimize animal discomfort and to sacrifice the fewest animals. All experiments were done in accordance with the guidelines established by the Animal Care Committee of Fourth Military Medical University.

All the rats were anesthetized with an intraperitoneal injection of pentobarbital sodium (50–60 mg/kg), and then the back region was shaved and aseptically prepared with iodophor. A laminectomy was performed at the T8 level to expose the cord underneath without disrupting the dura. After the spinous processes of T7 and T9 were clamped to stabilize the spine, the exposed dorsal surface of the cord was subjected to compression as described before [42]. Briefly, a compression plate attached to a 20 g copper rod was used. The compression plate was lowered down ventrally at a rate of 0.5 mm/min. It reached the bottom 5 min later and was removed at a constant speed.

After compression injury, 24 rats were used for RT-PCR assay to detect TLR4 mRNA expression at 0 h, 3 h, 24 h, 72 h, 7 d, and 14 d after injury. Another batch of rats was used for MyD88 blockade observation (Table 1). Locomotor functions were evaluated, and histological analyses were performed on this batch of animals.

### 2.2. Reverse Transcription PCR (RT-PCR) for TLR4

TLR4 mRNA levels after SCI were detected by using RT-PCR. After compression SCI as previously described, rats were anesthetized and sacrificed at 0 h, 3 h, 24 h, 72 h, 7 d, and 14 d after compression injury (for every time point, *n* = 4). Total RNA was isolated from 10 mm of thoracic spinal cord tissue centered at the injury site. RNA was purified using the Trizol reagent according to the manufacturer's instructions. In order to remove any traces of contaminating DNA, RNA samples were treated using the RNase-free DNase I (TaKaRa, Co., Ltd. Dalian, China). One microgram of total RNA was reverse transcribed into first strand cDNA in each 20 *μ*L reaction mixture, according to the manufacturer's instructions (TaKaRa, Co., Ltd. Dalian, China). Primers used were as follows: TLR4, sense: 5′-GTTGGATTTTACGAATTCCACCT-3′, and antisense: 5′-TGCTTCTTGTTCTTCCTCTGATG-3′; the expected size was 646 bp; *β*-actin, sense: 5′-GAGAGGGAAATCGTGCGTGAC-3′, and antisense: 5′-CATCTGCTGGAAGGTGGACA-3′; and the expected size was 453 bp.

PCR was performed according to the manufacturer's instructions. The PCR mixture (20 *μ*L) contained 10 *μ*mol of each primer, 200 *μ*M dNTPs, 25 mM MgCl_2_, 2 *μ*L 10x PCR buffer, 1 U Taq DNA polymerase (TaKaRa, Co., Ltd. Dalian, China), and 10 *μ*M purified cDNA. Denaturing, annealing and extension times were 30 seconds each, at 95, 57, and 72°C, respectively. cDNA samples were amplified for 40 cycles. The specific product was separated on a 2% agarose gel (Biowest, Spain) and detected after staining with ethidium bromide under UV illumination.

### 2.3. MyD88 Blockade Experiments

After compression injury as described previously, rats were randomly divided into two groups: MIP group and CP group. Each rat of MIP group received intramedullary injection of 5 *μ*L MyD88 inhibitor peptide (MIP, 100 *μ*mol/L, Imgenex, USA), and each rat of CP group was given 5 *μ*L control peptide (CP, 100 *μ*mol/L, Imgenex, USA), dissolved in 0.01 mol/L PBS, immediately after compression injury at the injury site. The peptides we used for inhibition and control contain a protein transduction (PTD) sequence (DRQIKIWFQNRRMKWKK) derived from antennapedia, and the CP consists of only the PTD sequence, which renders the peptide cell permeability. The injections were done at both sides of the dorsal vein at a constant rate of 1 *μ*L/min at about 1 mm below the spinal cord surface. Then the muscles and skin were closed in layers, and every two rats were placed in one cage. Manual bladder expression was performed at least twice per day until reflex bladder emptying was established.

#### 2.3.1. Histological Analysis

Animals were perfused via the left cardio-ventricle with 100 mL of physiological saline and subsequently with 400 mL of 4% paraformaldehyde in 0.1 M phosphate buffer (PB, PH = 7.4) at 7 d and 14 d, respectively (*n* = 5). Three centimeters spinal cord was carefully dissected out with the injury site in the center and was immersed in 25% sucrose solution for 24–48 h at 4°C until it sank. Sagittal sections were cut at 20 *μ*m in thickness with a cryostat and thawed-mounted onto gelatinized slides. The sections were kept at −20°C until used.

 For Haematoxylin & Eosin (H&E) staining, sections were washed briefly in distilled water, stained in haematoxylin solution for 5 min. After this, sections were washed in running tap water for 5 min and differentiated in 1% acid alcohol for 30 sec. Then sections were washed in tap water for 1 min, placed in eosin for 30 sec, dehydrated through 70%, 80%, 90%, and 100% alcohol 2 min each, clear in 2 changes of xylene, 5 min each, and mounted with xylene-based mounting medium.

For IHC staining, the sections were rinsed in PBS 3 times 5 min and blocked with 1% bovine serum albumin for 1 h to eliminate nonspecific staining and then incubated with primary antibodies 16–24 h at room temperature. Primary antibodies were rabbit anti-Cleaved Caspase-3 (1 : 400, Cell Signaling Technology, USA), rabbit anti-GFAP (1 : 4000, Sigma, USA), mouse anti-NeuN (1 : 500, Sigma, USA), and goat anti-IgG (1 : 400, Millipore, USA). Then sections were rinsed with PBS 3 times, 5 min for each time, and incubated with the appropriate secondary antibody for 2 h at room temperature. Sections were examined on Olympus BX 51 microscope (Olympus, Japan) or FV 1000 confocal microscope (Olympus, Japan).

For cell counting, five sections were chosen for analysis in each rat, namely, the section with central canal and two adjacent sections on both sides. After pictures were taken, images were opened in Adobe Photoshop 9.0 and stuck together as a montage. Caspase-3-positive cells (with nucleus indicated by Hoechest staining) were counted from 4 represented areas randomly chosen in the sections. NeuN-positive cells within the first and second 1000 *μ*m distance to the lesion border were counted. The mean values of the five sections in each rat were used for statistical analysis. For lesion area measurement, GFAP staining images were taken as mentioned previously. The boundary of the injured area was outlined according to the differences between normal tissue and necrotic tissue or cavity. The pixels of the outlined area were calculated and then converted into the size of injury by using Adobe Photoshop 9.0 software.

#### 2.3.2. Western Blot Assay

At 7 d after compression SCI, animals were deeply anesthetized and sacrificed (*n* = 5). One-centimeter long spinal cord tissues, 5 mm rostral and 5 mm cordal to the injury site, were removed rapidly and stored in liquid nitrogen and then processed for extraction of protein. Briefly, tissue samples were homogenized with 0.5 mL of ice-cold lysis buffer (20 mM Tris-HCl, pH 7.5, 1 mM EDTA, 5 mM MgCl_2_, 1 mM DTT, 20 *μ*g/mL aprotinin, 1 mM PMSF, and 2 mM sodium orthovanadate). The homogenates were centrifuged at 13,000 rpm for 10 min at 4°C and supernatant were removed. The protein concentration was determined using Bradford method, a detergent-compatible protein assay with a bovine serum albumin as standard. Samples were boiled at 100°C for 10 min and then were electrophoresed on 10–15% SDS-PAGE and transferred onto a nitrocellulose membrane (Millipore, Bedford, MA, USA). The filter membranes were blocked with 5% nonfat milk for 1.5 h at room temperature and incubated with the primary antibody (phospho-p38 MAPK 1 : 1000, Cell Signaling Technology, MA, USA; caspase-3, 1 : 1000, Cell Signaling Technology, MA, USA; TNF-*α* 1 : 500, Santa, CA, USA; IL-1*β*, 1 : 1000, gifted by Professor Beifen Shen, Department of Molecular Immunology, Beijing, China) 16–24 h at 4°C. Then the membrane was washed with TBST buffer and incubated with the secondary antibody conjugated with horseradish peroxidase (1 : 8000; Jackson ImmunoResearch, USA) for 1 h at room temperature and visualized in ECL solution. The density of specific bands was measured with Image J (NIH, USA) software.

#### 2.3.3. BBB Score

BBB scales were used to detect the recovery of the motor function every day from 0 d to 14 d after injury. Each rat was placed in an 80 × 130 × 30 cm open field and run for 4 minutes and scaled from 0 to 21 point according to the guidelines [43].

#### 2.3.4. Beam-Walking Tests

 Beam-walking tests were used to detect the distance from the rump to the plane to reflect the recovery of the motor function at 7 d and 14 d after injury. The apparatus used for behavioral analysis beam is 120 cm long and 12 cm wide and supported by a 70 cm framework. We set up a video camera and recorded the animal's behavior walking from one end to the other end of the beam. Then we did video frame extraction and selected 5 represented frames to measure rump height by using Image tool 2.0 (UTHSCSA) software.

#### 2.3.5. Footprint Analysis

 Footprint analysis was conducted at 7 d and 14 d after injury. A piece of white paper (15 × 89 cm^2^) was put on the track and both of the hindlimbs plantar surfaces were colored blue and dorsal surfaces red [44]. Then the rats were allowed to walk from one end of the track to the other end. When foot dorsal surface touched the track, the white paper would be printed with red color, while when foot plantar touched the track, blue color would be left on the white paper. Then the whole paper with prints was scanned for analysis. Using Adobe Photoshop 9.0 software, the percentage of red pixels defined as red/(blue + red) × 100% was calculated to reflect the condition of toe dragging. Stride length (distance between the centers of ipsilateral adjacent footprints) and stride width (perpendicular distance between the centers of left and right hind limbs) were measured, and the average of five steps in each case was used for statistical comparison. Because only when injured rats walk well with plantar step, stride length and width could be clear enough to be measured, these parameters were taken only at 14 d after injury.

### 2.4. Statistical Analysis

 All data were presented as mean ± standard deviation. The statistical significance of differences between groups was determined by one-way ANOVA followed by Tukey test. The statistical program OriginPro 7.0 for windows was used for statistic analysis. Significant levels were set at *P* < 0.05 or *P* < 0.01.

## 3. Results

### 3.1. Expression of TLR4 mRNA after Compression SCI

**Figure 1:**
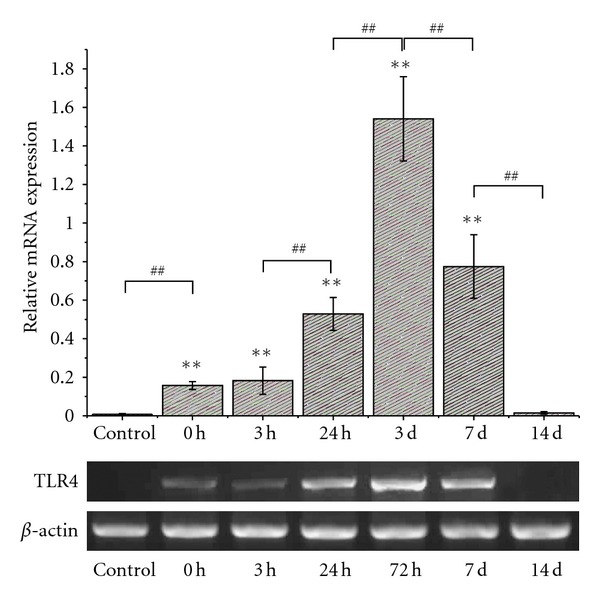
Semiquantitive analysis of TLR4 mRNA expression at each time point after spinal cord injury by RT-PCR. Levels of TLR4 expression were presented as the ratio of the area integral values under absorption curve of TLR4 with the area integral values of *β*-actin. **Compared with the control group *P* < 0.01; ^##^compared with the previous group *P* < 0.01.

RNA extracted from spinal cord tissue after SCI at different times revealed time-related changes of TLR4 mRNA expression.TLR4 mRNA were dramatically increased at 24 h after SCI, with peak expression evident by 72 h after injury, and it was lowered to normal level at 14 d after injury (Figure 1).

### 3.2. Injury Area of Primary Injury

**Figure 2:**
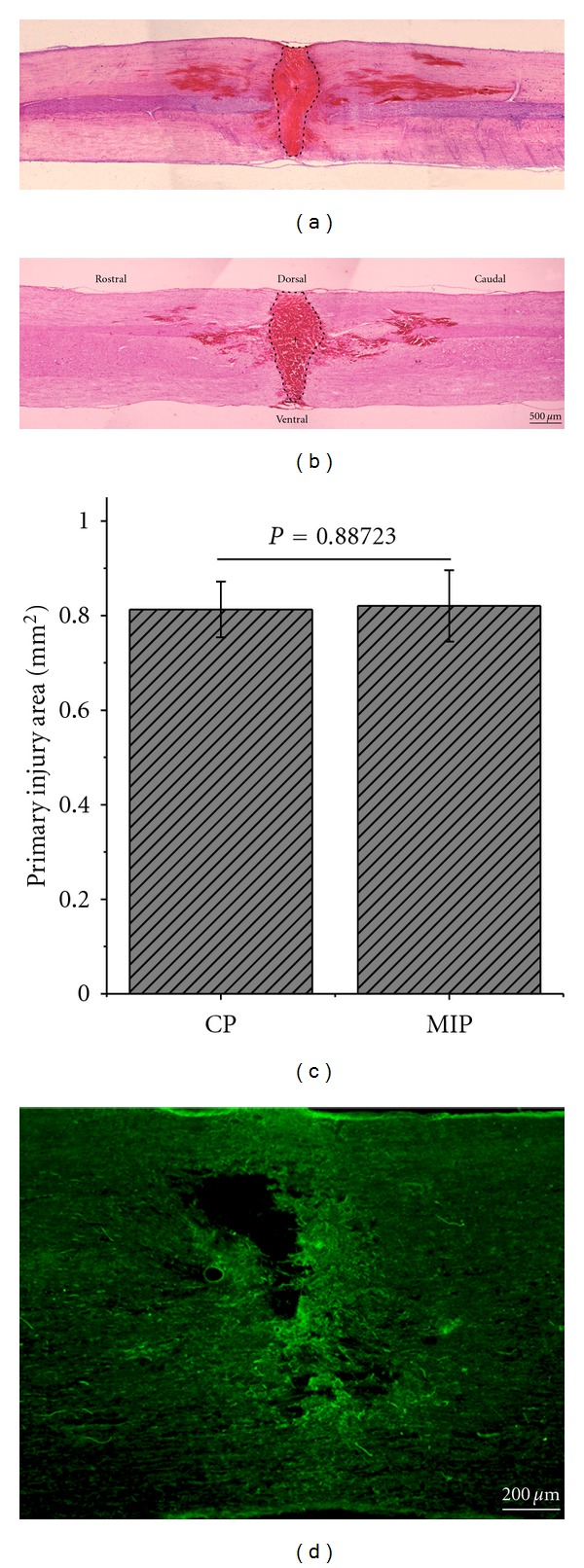
Injury area of primary injury. The boundary of injury area was dot-lined in CP (a) and MIP (b) group. The cross indicated the epicenter of the lesion. (c) is the histogram of the injury area in CP and MIP groups. There was no significant difference between the two groups in primary injury (*P* = 0.88723). Scale bar = 500 *μ*m. (d) IgG immunoreactivity in the spinal cord immediately after injury. Scale bar = 200 *μ*m.

HE staining showed that there was no significant difference in injury area immediately after compression injury between the two groups. In CP group, injury area was 0.8127 ± 0.05905 mm^2^, and in MIP group, it was 0.8206 ± 0.07541 mm^2^ (Figure 2(c), *P* = 0.88723).

### 3.3. Inflammatory Cytokines and p38 MAPK Activation

**Figure 3:**
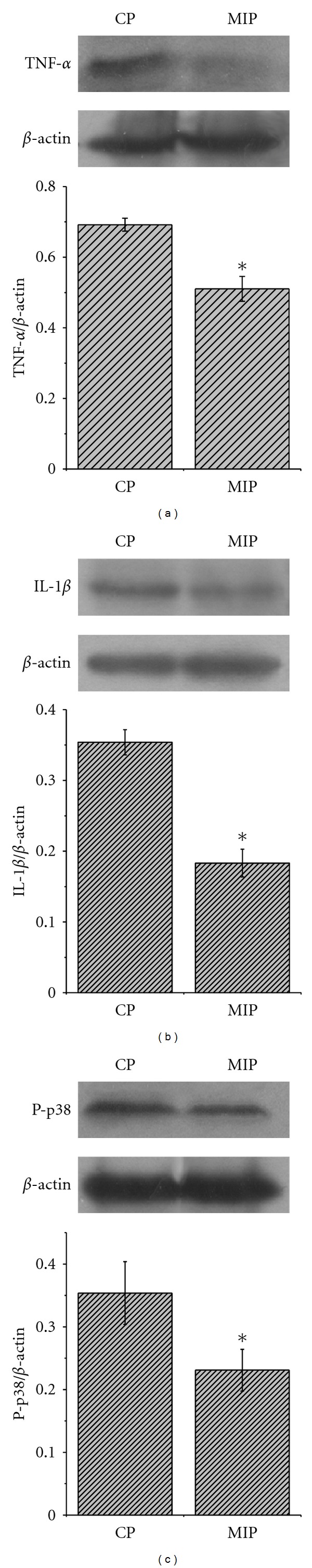
Western blot analysis for proinflammatory cytokines and phospho-p38 MAP kinase. TNF-*α*, IL-1*β*, and P-p38 MAP kinase proteins in the spinal cord were detected at 7 d after compression SCI in both MIP- and CP-treated groups. Levels of TNF-*α* (a), IL-1*β* (b), and P-p38 MAP kinase (c) were significantly decreased in MIP group, respectively, compared with CP group. Levels of proteins expression were presented as the ratio of the band area integral values under absorption curve of each protein with the area integral value s of *β*-actin. The quantified data are represented 4 animals per group (*P < 0.05).

Western blot assay showed that inflammatory factors were reduced by MIP treatment at 7d after compression injury in the spinal cord. In MIP group, TNF-*α* was dramatically decreased, compared with that in CP group (Figure 3(a), *P* < 0.05). And IL-1*β* in CP group was about twofolds of that in MIP group (Figure 3(b), *P* < 0.05). Phospho-p38 MAPK, which is associated with TLRs MyD88-dependent pathway, was also reduced after given MIP (Figure 3(c), *P* < 0.05).

### 3.4. Cell Apoptosis and Injury Areas

**Figure 4:**
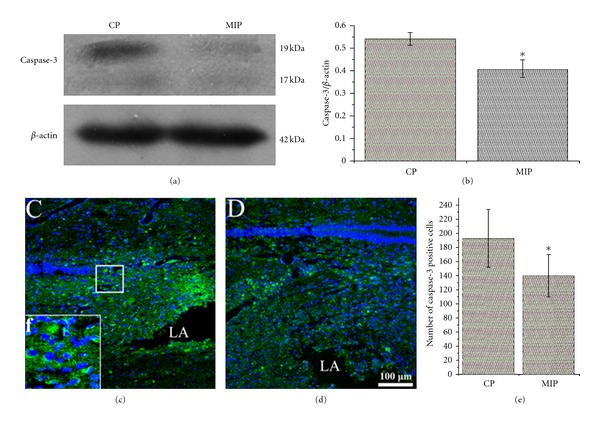
Total apoptosis at 7 d after injury. Western blotting (a) showed that, compared with CP group, caspase-3 in the spinal cord was significantly decreased in MIP group. Levels of proteins expression were presented as the ratio of the bands area integral values under absorption curve of each protein with the area integral values of *β*-actin. The quantified data (b) are represented, for each group, *n* = 5, *P < 0.05. Immunohistochemistry for caspase-3 (green) and Hoechst 33342 (blue) counterstaining showed more apoptotic cells in CP group (c) than that in MIP group (d) regardless of cell types, and (f) was the higher magnification of the square in (c) showing that caspase-3 immunoreactive product appears in cytoplasm of the cells. The diagram (e) showed number of caspase-3 positive cells counted from 4 represented areas randomly chosen in the sections, and the number of apoptotic cells in MIP group was significantly decreased, compared with CP group (*P < 0.05).

Western blot analysis was also performed to detect total caspase-3 (Figure 4(a)), a marker for the apoptosis. The result showed that in MIP group caspase-3 was dramatically decreased, compared with which in CP group (Figure 4(b)). To confirm the result, caspase-3 IHC was done on the spinal cord sections at 7 d after surgery (Figures 4(c) and 4(d)). Cell counting showed by randomly choosing 4 represented areas that the number of caspase-3 positive cells was significantly decreased in MIP group (139.1 ± 29.8) compared with that in CP group (192.0 ± 41.0) (Figure 4(e)).

**Figure 5:**
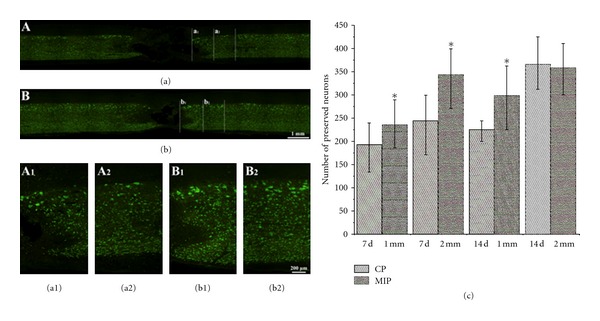
NeuN-positive cells in the spinal cord at 7 d and 14 d post injury. (a) and (b) are the representative pictures of NeuN immunohistochemical staining in CP group and MIP group, respectively, at 14 d. The areas of a_1_ and b_1_, are the first 1000 *μ*m from the lesion border indicated by initiating appearance of NeuN positive cells, while a_2_ and b_2_ are second 1000 *μ*m adjacent to a_1_ and b_1_ respectively; and A_1_, A_2_, B_1_, and B_2_, are the magnifications of a_1_, a_2_, b_1_, and b_2_ respectively. (c) As compared to the CP group, the number of neurons is significantly higher in the MIP group in the first 1000 *μ*m and the second 1000 *μ*m at 7 d after injury (*P < 0.05), and at 14 d there was an obvious difference between CP and MIP groups in the first 1000 *μ*m while there was no difference between these two groups in the second 1000 *μ*m.

Five sections of each group were used to do the NeuN IHC staining to detect the number of preserved neurons at 7 d and 14 d after injury, pictured by Olympus BX51 (Figure 5). NeuN-positive cells in 2000 *μ*m beside injury area at the two end stumps were counted. At 7 d after injury, the result showed that there were more preserved neurons in MIP group in the first (234.8 ± 49.2) and second (343.0 ± 60.3) 1,000 *μ*m compared with CP group 191.6 ± 47.3 and 244.4 ± 84.2 respectively (*P* < 0.05). At 14 d after injury, the results showed that MIP group had more neurons (296.9 ± 64.8) than that of CP group (224.8 ± 17.9) in the first 1,000 *μ*m area (Figures 5(a_1_, b_1_, A_1_, B_1_) (*P* < 0.05). While in the next 1,000 *μ*m area, there was no significant difference between the two groups (Figure 5(a_2_, b_2_, A_2_, B_2_) (*P* = 0.77719).

**Figure 6:**
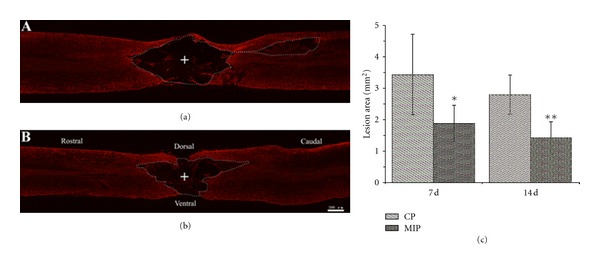
GFAP immunohistochemical staining in the spinal cord section at 7 d and 14 d after injury. (a) and (b) represent pictures of GFAP-stained sections of spinal cord in CP and MIP groups at 14d after injury, respectively. The boundary of the lesion area was dot-lined, according to the GFAP immunoreactive product. The cross indicates the epicenter of the lesion. (c) is the histogram of the lesion area in CP and MIP groups. There was a significant difference between the two groups at 7 d (*P < 0.05) and 14 d after injury (**P < 0.01). Scale bar = 200 *μ*m.

We used GFAP, a marker of the astrocyte, to show the injury area at 7 d and 14 d (Figure 6(a) and 6(b)) after injury. Statistics (Figure 6(c)) showed that there was a significant difference between the two groups in size of the lesion area. MIP group got a smaller injury area at 7 d (1.9 ± 0.6 mm^2^) and 14 d (1.4 ± 0.5 mm^2^) compared with that of CP group 3.4 ± 1.3 mm^2^ (*P* < 0.05) 2.8 ± 0.6 mm^2^ (*P* < 0.01), respectively.

### 3.5. Locomotor Recovery

During the whole process, three tests including BBB score, beam-walking test, and footprint analysis were performed to compare the recovery of the motor function in different treatments after SCI.

#### 3.5.1. BBB Score

**Figure 7:**
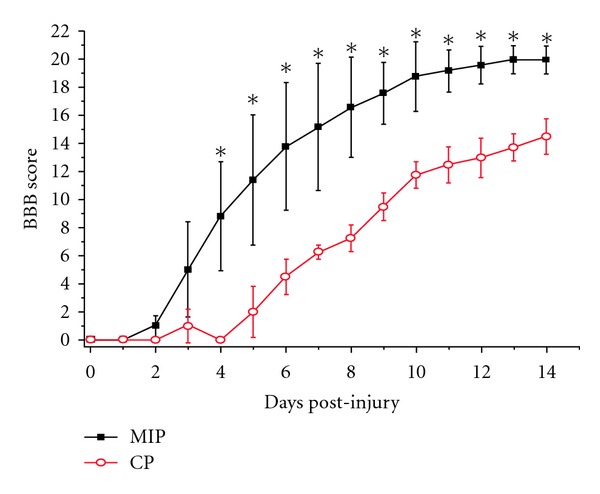
The statistical analysis of the BBB score at different time points between the CP and MIP group. Compared with the CP group, the BBB scores were significantly improved in MIP group at the fourth day after injury till the end of observation (14 d) (*P < 0.05).

BBB test was performed every day after injury. Results (Figure 7) showed that there was no difference in the first 3 days, while at the 4 d after injury, the rats in MIP group had better hindlimbs motor function recovery than those in CP group. At 7 d, rats in MIP group got an average score of 15.2 ± 4.6, which meant that those rats could support their body weight by the hindlimbs and sometimes walk coordinately. While CP group scored 6.3 ± 0.5, which meant that the rats could only move two or three joints of the hindlimbs extensively. At 14 d, rats in MIP group got an average score of 20.0 ± 1.0, which meant that the rats walked coordinately with tail consistently up and had no toe dragging. In CP group rats scored 14.5 ± 1.3 (*P* < 0.05), Which meant the rats could support its weight by the hindlimbs and walk coordinately with the tail down at this time.

#### 3.5.2. Beam-Walking Test

**Figure 8:**
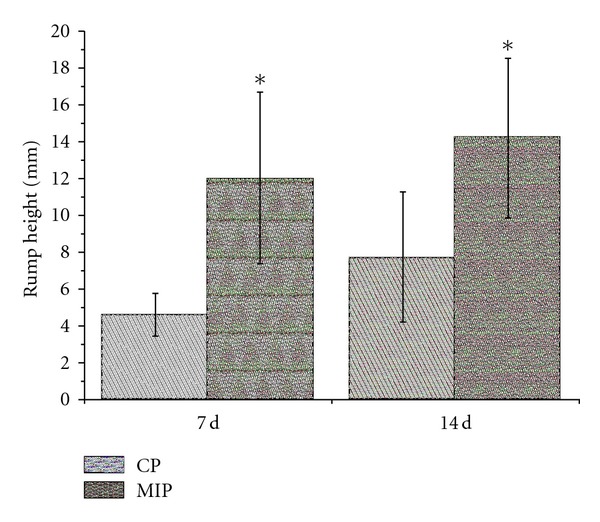
The rump height measured through beam-walking test in the CP and MIP groups. The body supporting ability recovered slightly with time, indicated by the rump height elevating within each group. Compared with the CP group, the rump height was significantly elevated in MIP group at 7 d and 14 d after injury (*P < 0.05).

 Beam-walking test was done to detect the rump height which means the vertical distance from base end of the rat tail to the surface of the beam. This distance reflects the ability of the rat hindlimbs to support its body weight. At 7 d and 14 d after injury there were significant differences in rump height between the two groups (Figure 8). The height was 12.0 ± 4.7 mm in MIP group and 4.6 ± 1.2 mm in CP group at 7 d (*P* < 0.05). At 14 d after injury, the height was 14.2 ± 4.3 mm in MIP group and 7.7 ± 3.5 mm in CP group (*P* < 0.05).

#### 3.5.3. Footprint Analysis

**Figure 9:**
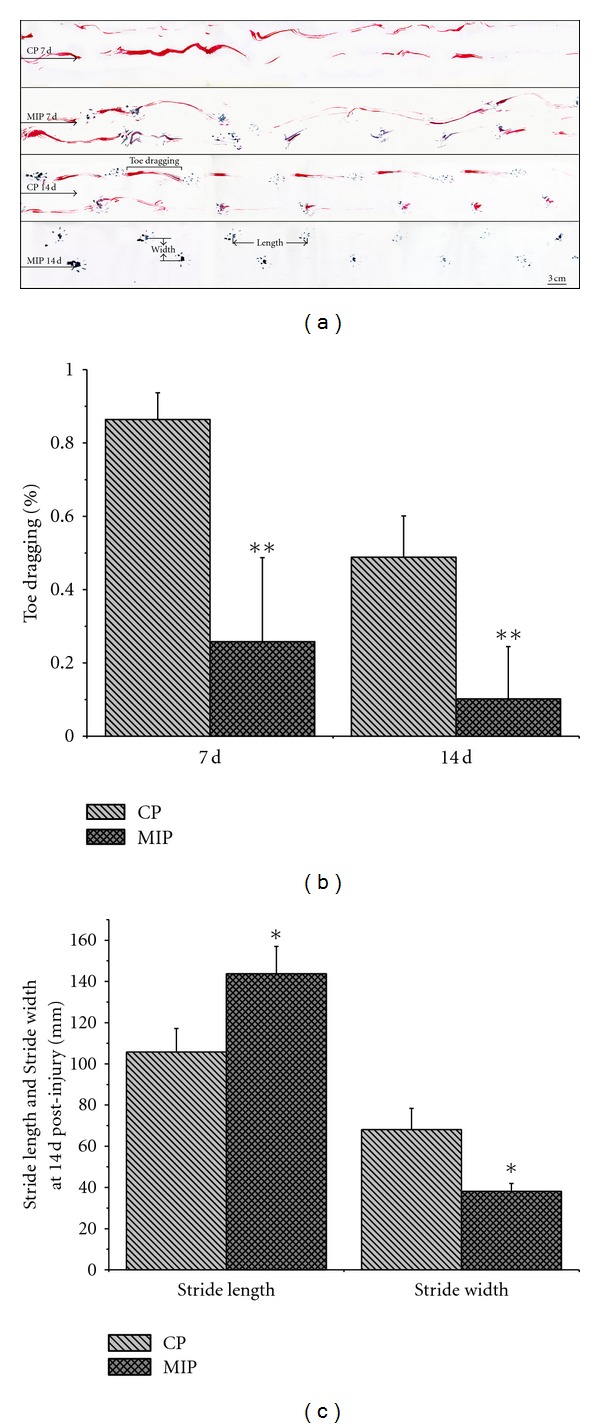
Footprint analysis at 7 d and 14 d after injury. (a) Foot prints of red and blue traces. Arrows point to the direction of walking. CP 7 d: there was only toe dragging, but no plantar stepping appeared. MIP 7 d: plantar prints appeared. CP 14 d: plantar prints were frequent and coordinated while toe dragging was also seen. MIP 14 d: the animal resumed almost normal walking. Scale bar = 3 cm. (b) Percentage of the toe dragging was significantly reduced in MIP group, compared with CP group at 7 d, 10 d, and 14 d (**P < 0.01). (c) Stride length at 14 d was significantly increased in MIP group in comparison to CP group (*P < 0.05); meanwhile the stride width was decreased in MIP group compared with CP group (*P < 0.05).

Three parameters including hindlimbs stride length, stride width, and the ratio of toe dragging were taken from the footprint analysis (Figure 9(a)). These measurements imply the gait recovery of the rats from SCI.  Figure 9(b) showed that at 7 d after injury, the percentage of red color pixels in MIP group was 25.8 ± 22.8%, and in CP group it was 86.4 ± 7.3% (*P* < 0.01); at 14 d, the percentage was 10.2 ± 14.3% in MIP group and 48.8 ± 11.3% in CP group (*P* < 0.05). Stride length and width (Figure 9(c)) measured at 14 d after injury indicated significant differences between MIP and CP treatments. Compared with CP group, MIP group had a bigger stride length (143.7 ± 13.3 mm) and smaller stride width (38.1 ± 3.9 mm) than that of CP group (105.7 ± 11.5 mm, 68.1 ± 10.3 mm; *P* < 0.05).

## 4. Discussion

Although whether endogenous ligands really trigger TLRs signals without pathogen is debatable [45], many studies have documented that TLRs responded to the host antigens as well as bacteria [32, 46, 47]. These DAMPs combine to TLRs to induce MyD88-dependent pathway to produce proinflammatory cytokines [15, 16, 22, 48]. Our previous study in vitro also showed homologous IgG induced microglia TLR4 expression and the TNF-*α* production upregulated [49].

In the injured area of the spinal cord, the blood-spinal cord barrier was destroyed by the primary injury, serum proteins were extravasated easily into the parenchyma of the spinal cord, as indicated by the IgG immunoreactivity, tissue disruption would produce sufficient debris, and injury may lead heat shock protein release from damaged neurons and serve as DAMP as well, all of which may activate TLRs signaling. In the present study, pathogen infection may not be excluded but was minimized by keeping dura intact and sterile manipulation. There was great amount of cell debris caused by compression in the local spinal cord, and serum proteins could easily enter to spinal parenchyma since the local blood-spinal barrier was seriously damaged. All these debris and serum proteins could serve as DAMP to bind and activate TLRs in microglia and other cells around the wound.

As one of the well-studied TLRs that could be triggered by endogenous DAMP, TLR4 has been documented to be involved in a vicious cycle which mediates neurodegeneration [50]. The other TLRs might also play roles in spinal cord injury-induced innate immune reactivities; nevertheless alteration of TLR4 expression may reflect TLRs responses to DAMP in the present study. In our experiment, TLR4 mRNA was upregulated 3 h after compression injury, peaked between 24 h and 72 h after injury, and then came back to the baseline at 14 d (Figure 1), according to RT-PCR detection. These data indicated that TLR4 responded to the SCI quickly, and its expression was increased at early period post injury. With this sterile SCI compression model, we also revealed by immunofluorescent staining that TLR4 protein was mainly expressed in microglial cells in the injured area of spinal cord (data not shown), indicating that the immune response associated with TLR4 in the early stage was born by microglial cells, which was the same result as Kigerl et al. reported [27]. According to the report, most TLRs were expressed twofolds more than sham injury controls as early as one day after spinal cord injury. Then two pathways of TLRs signaling would be activated as the DAMP combined with them. At early phase of TLRs activation, MyD88-dependent pathway may exert its inflammatory role dominantly via NF-*κ*B [31, 34].

After TLRs MyD88-dependent pathway was activated, it will mediate the expression of proinflammatory genes and leukocyte recruitment after CNS injury [51]. The present study obtained the similar results from the compression SCI in the rats. SCI caused IL-1*β* and TNF-*α* protein increased in the spinal cord tissue, as shown by Western blotting assay.

MyD88 is a common molecule shared by most TLRs (except TLR3) to activate NF-*κ*B, resulting in the production of large amounts of cytokines, including IL-1 and TNF-*α* [52, 53]. The levels of IL-1*β* increase rapidly after traumatic SCI [54–56]. TNF-*α* expression at both mRNA and protein levels is also an immediate event after SCI [57, 58]. TNF-*α* has been regarded as a key inflammatory regulator that can induce further cytokine production, inflammation, gliosis, demyelination, blood-brain-barrier damage, and cell adhesion [59]. Sharma et al. reported that TNF-*α* antiserum could alleviate the microvascular permeability disturbances, cell damage, and edema in the spinal cord trauma in the rat [60]. Nesic et al. reported that given IL-1 receptor antagonist at the site of injury could dramatically reduce the contusion-induced apoptosis and caspase-3 activity in the model of contused spinal cord injury [61].

In the context of proinflammatory cytokines production, cell apoptosis developed in the adjacent area to the injury site, indicated by caspase-3 elevation in the present study. These findings are identical to the previous report by Pais et al. [62] who demonstrated in vitro that necrotic neurons could activate cultured microglia to produce proinflammatory cytokines and thus induced apoptosis of hippocampal neurons. Therefore, inflammation at the early stage exacerbates the disruption at the injured tissue by enhancement of cell lost. Here we documented that traumatic injury to the spinal cord itself leads to the early inflammation by triggering TLRs MyD88-dependent pathway.

The peptides we used for inhibition and control contain a protein transduction (PTD) sequence (DRQIKIWFQNRRMKWKK) derived from antennapedia, and the CP consists of only the PTD sequence, which renders the peptide cell permeability. In the present study, these peptides were given immediately after the compression injury was performed. Although the effective duration of the MIP was not determined in our study, it may start approximately 24 h after administration as in vitro study MIP need, to be incubated with cells 24 h ahead of intervention. The blockade period could be partially overlapped with the main part of the upregulated TLR4 expression period indicated by RT-PCR data (Figure 1), especially to the early stage of the TLR4 MyD88 signals.

 In our experiment, inhibition of MyD88-dependent pathway with MIP significantly lowered the levels of TNF-*α* and IL-1*β*, and MIP treatment also inhibited the p38 MAPK activation in the injured spinal cord. Namely, blockade of MyD88 signal inhibited NF-*κ*B pathway and therefore downregulated the production of proinflammatory cytokines in the spinal tissue.

Meanwhile, cell apoptosis was reduced significantly by MIP treatment, indicated by caspase-3 expression. Cell counting for NeuN-positive cells also formed that more neurons survived in the MIP-treated group after SCI. These data are consistent with previous report by Liebermann and Hoffman that MyD88 mediates negative growth control, including growth suppression and apoptosis [63].

Amelioration of apoptosis resulted in more cells survival; at least more neurons were conserved, according to the NeuN-positive cell counting in the present study. As it well known, spared axons are pivotal for the functional recovery; more neurons survival means that more axons in the spinal cord are spared thus to enhance the function recovery. The behavioral tests indicated clearly the improvement of locomotor recovery in the rats treated with MIP, compared with the CP-treated group. The obvious improvement was seen from the 4th day after SCI till the end of our observation (14 d post injury). These data imply the protective effect of blockade of MyD88-dependent signaling pathway at early time in our experimental system.

We also found injury area smaller in the MIP group than that in the control group. These findings imply that lowered inflammation could preserve more cells including neurons and thus may contribute to less secondary injury and better behavioral function after SCI. Therefore, it would be protective to block TLRs MyD88-dependent pathway which maybe play an important role in the development of secondary spinal cord injury.

Proinflammatory factors of production cells may have totally reverse action depending on when the cells were activated and where they migrated [14]. Activated microglial cells can secret lots of products that have harmful effects on adjacent neurons, while it can also produce some beneficial factors such as BDNF, NT-3, and TGF-*β* [64–67]. However, some of these neurotrophic factors have been shown to induce apoptosis of oligodendrocytes or neurons, sometimes during developmental regulation of neuronal proliferation, sometimes in the injured adult organism [68]. Up to now, the question of two-edge sword is unsolved. It is generally accepted that suppression or inhibition of CNS macrophages at the early time is neuroprotective after SCI [69–73], though some authors hold that macrophages are beneficial to the recovery of SCI due to their anti-inflammation role [74, 75]. The latter point of view maybe result from the reduced accumulation of proinflammatory cytokines, neurotoxins, and proteases, all of which can be released by CNS macrophages [76–78].

Since the inflammation could be triggered by tissue disruption via TLRs activated by endogenous DAMPs, roles of TLRs in SCI have been studied extensively with many technologies. Using genetic knockout mice, studies showed the alteration in the CNS repair caused by TLR2, TLR4 deficiency [27], which is convinced and complete. However, to some extent, it is different with our results. The reasons may lie in discrepancy of animal models, detailed experimental systems, and the time window of proinflammatory factors that affect the SCI process. Therefore, specific antagonist/inhibitor of MyD88-dependent pathway at specific phase only may target the harmful effects, protect the spared tissue after SCI regardless of the cell types, and promote the recovery through inhibition of inflammation and cell apoptosis.

It is interesting that stimulated astrocytes with TLRs ligands could inhibit their ability to uptake excess glutamate [79, 80]. As it is well known, SCI leads to the release of massive of glutamate [81]. And excitotoxicity of glutamate was widely studied and showed significant involvement in secondary damage [81]. In 1999, Vezzani et al. reported that glutamate could induce IL-1 synthesis [82]. These studies suggest that TLRs may be associated with not only inflammation but also many other mechanisms involved in spinal cord secondary injury.

In conclusion, the present study showed with rat compression SCI models that traumatic injury that caused tissue damages may trigger TLRs signals, and TLRs MyD88-dependent pathway may play crucial roles in the secondary injury. Inhibition of the TLRs MyD88-dependent pathway at early time after spinal cord injury could protect the spinal cord from inflammation and apoptosis and promote the functional recovery.

## Figures and Tables

**Table 1:** Animal assignment for MyD88 blockade experiments.

	Total	Histological analysis			Western blot assay
		0 h	7 d	14 d	7 d
CP	18	3	5	5	5
MIP	18	3	5	5	5
